# The Time-Course of Visual Categorizations: You Spot the Animal Faster than the Bird

**DOI:** 10.1371/journal.pone.0005927

**Published:** 2009-06-17

**Authors:** Marc J.-M. Macé, Olivier R. Joubert, Jean-Luc Nespoulous, Michèle Fabre-Thorpe

**Affiliations:** 1 Université de Toulouse, UPS, Centre de Recherche Cerveau et Cognition, Toulouse, France; 2 CNRS, CerCo, Toulouse, France; 3 Université de Toulouse, Octogone, Jacques Lordat, UTM, Toulouse, France; Ecole Polytechnique Federale de Lausanne, Switzerland

## Abstract

**Background:**

Since the pioneering study by Rosch and colleagues in the 70s, it is commonly agreed that basic level perceptual categories (dog, chair…) are accessed faster than superordinate ones (animal, furniture…). Nevertheless, the speed at which objects presented in natural images can be processed in a rapid go/no-go visual superordinate categorization task has challenged this “basic level advantage”.

**Principal Findings:**

Using the same task, we compared human processing speed when categorizing natural scenes as containing either an animal (superordinate level), or a specific animal (bird or dog, basic level). Human subjects require an additional 40–65 ms to decide whether an animal is a bird or a dog and most errors are induced by non-target animals. Indeed, processing time is tightly linked with the type of non-targets objects. Without any exemplar of the same superordinate category to ignore, the basic level category is accessed as fast as the superordinate category, whereas the presence of animal non-targets induces both an increase in reaction time and a decrease in accuracy.

**Conclusions and Significance:**

These results support the parallel distributed processing theory (PDP) and might reconciliate controversial studies recently published. The visual system can quickly access a coarse/abstract visual representation that allows fast decision for superordinate categorization of objects but additional time-consuming visual analysis would be necessary for a decision at the basic level based on more detailed representations.

## Introduction

“As soon as you know it is there, you know what it is” [1], “Sometimes you know it is there before you know what it is” [2], “Detecting objects is easier than categorizing them” [3]. The speed at which objects are detected and categorized had been a very controversial topic lately. But in all cases, scientists are using categorization at the basic or subordinate levels. Indeed, in the 70s, Rosch and colleagues proposed that among the different levels of categorization, organized as a hierarchical taxonomic system, one of them is accessed first whatever the perceptual modality used [4]. This so-called *basic level* is defined as the most abstract level where objects still share a common shape and could correspond to an optimum in terms of cognitive efficiency of categorization [5]. The primacy of the basic level (e.g, dog or chair) over the *superordinate* (e.g. animal or furniture) and *subordinate* levels (terrier or rocking chair) was further assessed in object naming and category membership verification experiments [4]. These basic level categories are also the easiest categories to be learned by children [6] and include the words most spontaneously used by adults in free naming of objects [4]. It was thus inferred that the basic level should correspond to the stored mnesic representation that is activated first when an object is perceived. Superordinate levels were then considered as abstract generalizations of basic level representations and subordinate levels as perceptually less inclusive categories. This idea has later been refined by Jolicoeur [7] and Murphy [5] who introduced the concept of *entry level category* to explain the shorter reaction times found at the subordinate level for some atypical members of basic categories. A penguin is categorized faster as a penguin than as a bird, because unlike sparrows or blue tits, its appearance is more distant from the prototypical bird. Entry level would normally be at the basic level but could be found at the subordinate level in some extreme cases.

The shift of entry level towards subordinate level and the loss of basic level advantage in some specific cases were also observed with increasing expertise [8], [9]. Bird and dog experts are equally fast to categorize these animals at the subordinate and basic levels and they frequently use the subordinate name of an object in their field of expertise whereas non-experts use basic level names. In fact, Grill-Spector and Kanwisher [1] claim that basic level categorization of an object and even object identification do not take more processing time than object detection; a claim recently challenged in visual detection tasks [2], [3]. However, these observations concerned only the speed of access to the basic and subordinate levels; very few studies have questioned the prevalence of the basic level over the superordinate level. The first element came from Murphy & Wisniewski [10], who reported that the speed advantage of basic over superordinate levels was reduced -although still present- when objects were presented in full scenes instead of the frequently used isolated objects on a neutral background. A second argument came from the literature on child development as Mandler et al. [11] showed that children 18–30 months old develop global conceptual animal and vehicle categories without clearly differentiating basic level categories within these domains. The implication is that in the development of hierarchical categorical systems, basic level categories would not form the entry level.

More recently, experiments on rapid visual categorization at the superordinate level definitely challenged the traditional view. The surprising speed at which subjects can detect animals or vehicles in natural scenes in a simple go/no-go categorization task raised many questions about the basic level dominance, at least in the visual modality [12], [13]. Cerebral activity differs between target and non-target trials from 150 ms onwards after stimulus onset. This temporal constraint already challenges most models of object recognition and VanRullen and colleagues [13] first pointed out the fact that it would be very difficult to expect human performance to be even faster for the basic level categorization. Contrary to the original experiments that used a reduced set of isolated object drawings, these latter studies involved trial-unique stimulus presentations as the very varied target natural scenes were presented only once. Moreover, subjects were asked to respond manually, as opposed to the otherwise frequently used verbal responses. In fact, all studies that reported a basic level advantage have involved, albeit to various degrees, some lexical-semantic processing! Yet, the linking of object visual representations with their names is, without any doubt, time consuming. Indeed, in the human medial temporal lobe, neurons were found that respond both to the picture of a celebrity and to its written name, but with long response latencies -around 300 ms at the earliest [14]. On the contrary, the use of the rapid visual categorization task reduces the need for lexical access. This is supported by experiments which have shown that macaque monkeys can perform the task [15], [16] with accuracy scores slightly below human scores (90% vs. 94% correct) but with considerably faster speed, as median reaction times are 150 ms shorter in monkeys than in humans [17]. Recently, Large et al. [18] used a yes/no visual categorization task and found a weak advantage (<15 ms) for superordinate level in visual categorization of isolated drawings of objects. However, this effect could result from a speed/accuracy trade-off, as subjects were 2% more accurate in the slower basic level categorization task. Moreover, as in most previous studies, they used repeatedly a relatively small number of isolated objects drawings (n = 146) so that memory effects could affect the results.

So it might be that Rosch's results on the basic level advantage did not apply to pure visual categorization. Different theories can account for the commonly observed basic level advantage. Visual stimuli would be categorized first at an entry “perceptual” level, before accessing more inclusive (superordinate) or specific (subordinate) levels [7]. Alternatively to such two-stage process, Murphy and Brownell [5] proposed a differentiation theory according to which all category representations would be activated in parallel. For them the “basic category” advantage would emerge from the fact that basic representations are optimally distinctive and informative. Finally, a Parallel Distributed Processing (PDP) theory was proposed by McClelland, Rogers and Patterson [19], [20] in which objects representations would be activated from broad to fine so that large categories would be activated before tuning to more specific representation. For these authors, the basic level advantage would emerge because whereas the word “bird” would be activated after the word “animal”, it would be activated much faster as generalization occurs faster when similarity between items is higher.

In the present study, we used the rapid visual go/no-go categorization task introduced by Thorpe and al. [12] to compare human processing speed when categorizing natural scenes at the superordinate level (animal/non-animal) or at the basic level (bird/non-bird or dog/non-dog). Natural scenes were briefly flashed and subjects were under strict instruction to “respond as fast and as accurately as possible” within one second. With such temporal constraints and the request of a manual response, performance could rely on perceptual representation with no interference of linguistic representations. Thus, early accurate response limited to a given stage of object processing would point towards the object perceptual representation that is accessed first. Following the PDP theory, the superordinate category should be accessed faster whereas the two other theories predict a faster access to the basic level category. To avoid any effect due to stimulus repetition, the tasks used numerous varied pictures that were seen only once by a given subject. The protocol allowed unbiased performance comparisons as, over the group of subjects, we compared the same sets of images classified either as animal, bird or dog. Moreover, to ensure that subjects categorize the stimuli only at the requested level in the basic level tasks (bird or dog), half of the non-targets in the first experimental series were images from the same superordinate category (non-bird or non-dog animals). The correlation between processing time and non-targets categories was further analyzed in a second experimental series by varying the proportion of animal non-targets.

## Materials and Methods

### Ethics statement

All experiments met the requirements of the COPE (Comité opérationnel pour l'éthique dans les sciences de la vie). All subjects volunteered and gave their written informed consent to participate in the experiment.

### Experimental series 1. Dogs and birds: superordinate versus basic categorization

#### Participants

Two groups of 18 subjects (9 women, 9 men) were tested in two experiments. In the bird experiment the mean age was 32 years (20–52); in the dog experiment the mean age was 31 (23–52). Five subjects were tested in both experiments. All subjects had normal or corrected-to-normal vision.

#### Procedure

**Figure 1:**
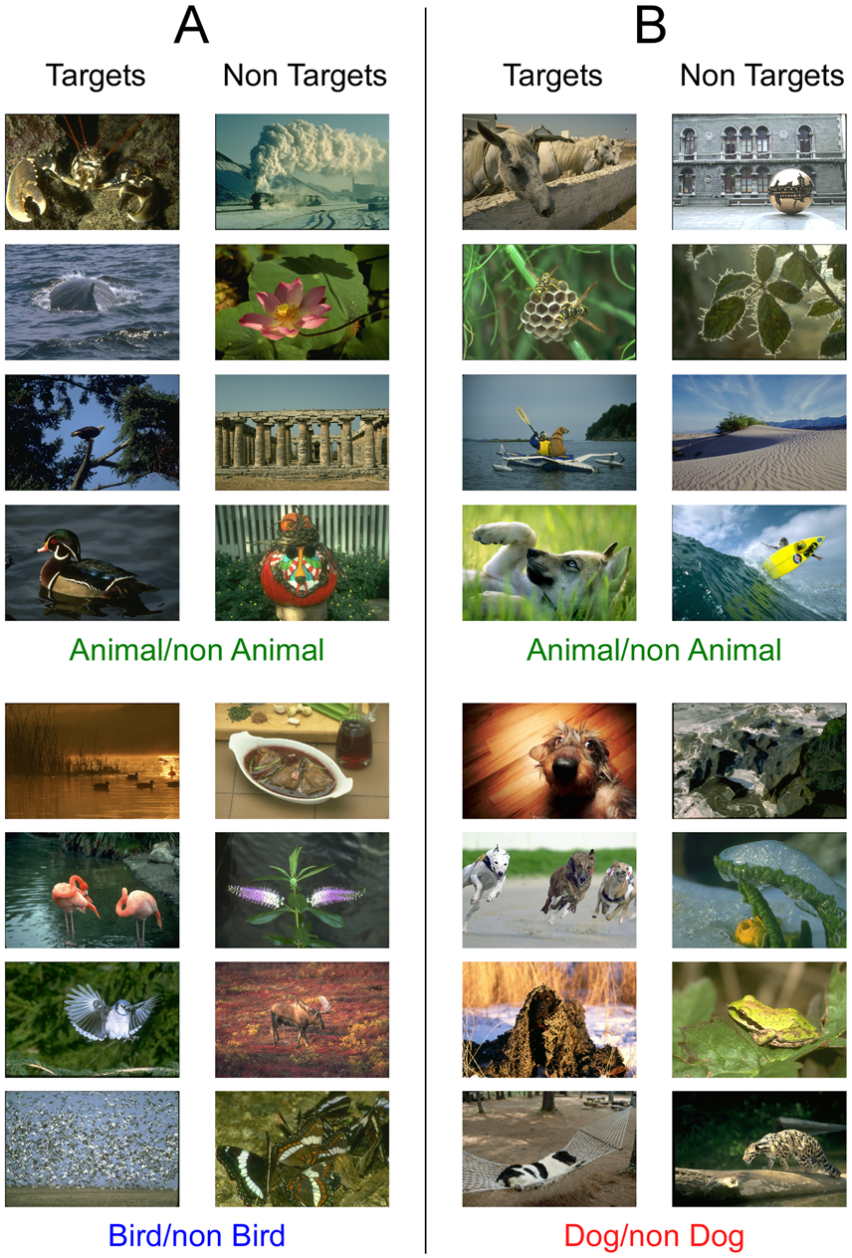
Examples of target and non-target images in the Bird experiment (A) and the Dog experiment (B) used either in the superordinate or in the basic level task. Note that in each experiment, half of the targets in the animal/non animal task are images of the corresponding basic level category (birds or dogs). In basic level tasks, half of the non-targets are images from the same superordinate category (non-bird or non-dog animals).

Subjects were seated at 1 meter from a computer screen in a dimly lighted room. They started the experiment by placing a finger over a response pad for at least one second. A fixation cross appeared for 300–900 ms, immediately followed by a photograph of a natural scene flashed in the centre of the screen for 26 ms (apparent size: 20°×13.5°). With non-target photographs, subjects had to keep pressing the button (no-go response). They were instructed to release the button (go-response) as quickly and accurately as possible when the scene contained a target (animal, bird or dog). They had 1 second to trigger their go response after which any response was considered as a no-go. The inter-stimulus interval time (ISI) was random in the range 1.6–2.2 s (mean: 1.9 s). Two experiments were run to compare human performance in superordinate and basic level tasks. In both experiments, the superordinate level task was an animal/non-animal (A/nA) categorization. At the basic level, subjects were required to perform either a bird/non-bird or a dog/non-dog (Fig 1) categorization. For a given experiment, a subject completed 16 blocks of 96 trials: 10 at the basic level (either bird or dog) and 6 at the superordinate level. A training block of 48 trials preceded each categorization task. To avoid any bias associated with learning, half of the subjects began with the superordinate task, the other participants started with the basic level task. As each image was only seen once by a given subject, the main concern was to avoid any bias induced by the selection of natural photographs for the tasks. In the superordinate A/nA task, half of the animal targets belonged to the basic category on which the subject was going to be tested (birds or dogs). Thus a series of 96 trials included 24 pictures of birds (or dogs) 24 pictures of all other animals and 48 neutral non-targets. In the basic level categorization task, half of the non-target photographs included non-bird or non-dog animals depending on the (bird or dog) target, while the other half were non-animal (neutral non-target) pictures. Thus, a series of 96 trials included 48 pictures of birds (or dogs), 24 neutral non-targets and 24 non-targets of the animal superordinate category. This protocol allowed image counterbalancing across conditions and subjects. All bird (dog) photographs were seen by different subjects as targets in the animal task or as targets in the bird (dog) task. Similarly, all images of non-bird (non-dog) animals were seen by some subjects as targets in the superordinate task and by others as non-targets in the basic level task. The neutral (non-animal) images were also used as non-targets either in the superordinate or in the basic level categorization tasks for different subjects. With such a protocol, performance at different levels of categorization can be compared on the same sets of images and the effects observed can be confidently attributed to task requirements and not to image bias. As personal expertise could play a role in shaping visual representations, none of the subjects included in the present study were bird (dog) experts.

#### Stimuli

Each experiment required a total of 1536 images, chosen to be as varied as possible from a Corel Database (Fig 1). All images of birds (n = 624) and dogs (n = 624) were seen once by each subject and processed either at the basic or the superordinate level. They contained a large range of species (birds of prey, parrots, sparrows, wading birds, gulls… or shepherds, mastiffs, poodles, spaniels, dachshunds…). They were presented in varied contexts at all scales from close-ups to far views. Birds were presented swimming, flying or resting in a variety of natural contexts, with man-made environment used only very rarely. Dogs on the contrary were presented in more varied scenes including urban outdoor or indoor contexts with or without humans and manmade objects. Other animal images (n = 384) were seen once by each subject either as a target at the superordinate level or as a non-targets in the bird (dog) basic task. They were also varied and could contain mammals, insects, fish, reptiles, etc. Subjects had no *a priori* knowledge about the size, position or number of target(s) in the pictures. “Neutral” non targets (n = 528) did not contain animals and were as varied as possible, including plants, flowers, buildings, people, man-made objects, various landscapes… All stimuli used in the present study can be seen at http://www.cerco.ups-tlse.fr/StimuliMace/ .

### Experimental series 2. Basic categorization: influence of varied non-target sets

#### Participants

14 subjects (3 women, 11 men) participated in this experiment with a mean age of 26 years (22–46). All subjects had normal or corrected-to-normal vision.

#### Procedure

In terms of stimulus presentation and subject's response, the protocol was the same as used in the first experimental series. Subjects were instructed to release the button (go-response) as quickly and accurately as possible when the scene contained a target. Targets were either animals or dogs. Each subject completed 12 blocks of 96 trials: 3 at the superordinate level and 9 at the basic level. When basic level categorization was required, three conditions -depending on the composition of the non-target set- were compared. As in the preceding experiments, the non-target set could include 50% of varied non-dog animals and 50% of neutral -non animal- photographs (Dog50%A). In the two extreme conditions, all non-targets were either non-dog animals (Dog100%A) or -non animal- neutral photographs (Dog0%A). A training block of 96 trials preceded each categorization task. All subjects began with the 3 blocks of superordinate categorization. After them, 6 subjects performed the 3 blocks Dog0%A that use the same neutral non-animal photographs than in the superordinate categorization task to allow direct comparison. Half of the remaining subjects started by Dog100%A and the other half by Dog50%A.

#### Stimuli

A set of 1152 images (504 dog pictures, 288 non-dog animal pictures and 360 neutral non-animal pictures) was used and all stimuli were as varied as in the first experimental series. Each dog picture was seen once -as target- by a given subject, in one of the four conditions (Superordinate, Basic 100, 50 or 0%A). All non-dog animal pictures were also seen once by a given subject, either as a target in the superordinate task or as a non-target in the 100 or 50%A basic level tasks. All neutral photographs were seen once as a non-target, either in the superordinate task, the 50 or the 0%A basic level tasks.

Across subjects, the protocol allowed each dog stimulus to be shown twice in the superordinate task and 4 times in each of the 3 basic categorization conditions.

With such a protocol, performance at the basic level of categorization can be compared on the same sets of dog-target images whereas the set of non-targets differs in its composition by the proportion of objects belonging to the same superordinate category. The effects observed can be confidently attributed to the composition of the non-target set and not to image selection bias. Here again none of the subjects included in the present study were dog experts to avoid any influence of special expertise with target stimuli.

## Results

Performance was evaluated using both accuracy and go-response reaction times (RT). Targets and non-targets were equiprobable in each series, which set the chance level at 50%.

### Experimental series 1. Dogs and birds: superordinate versus basic categorization

### Performance at the superordinate level (A/nA)

#### Accuracy

The control task was the A/nA superordinate task often used in previous studies [12], [13], [21]. A specificity of the present study was that half of the animal stimuli used as targets in the superordinate were either birds or dogs for better comparison with the performance on basic categorizations. The 50% other targets were very varied photographs of different types of animals. This experimental design allows us to analyze how the very varied set of selected bird (dog) pictures was processed at the superordinate level compared to all other animal pictures. The global accuracy in the control A/nA task was similar in both bird and dog experiments (95.8% and 95.5%, paired t-test, p = 0.71, t = 0.377). Bird pictures were categorized as “animal” with a slightly higher accuracy than non-bird animals (accuracy on targets: 98.5 vs. 94.8%; χ2, p<0.05; paired t-test, p<0.001, t = 7.751) whereas dog photographs were categorized as “animal” virtually with the same accuracy as non-dog animal pictures (accuracy on targets: 96.8 and 96.4%, paired t-test, ns). In both control tasks, subjects showed a tendency to be better at responding on animal targets (go responses) than at ignoring non-targets (no-go responses): Bird experiment: 96.6 vs. 95.0%; Dog experiment: 96.6 vs. 94.4% (in both cases, χ2, p<0.05; paired t-test, ns.).

#### Speed

**Figure 2:**
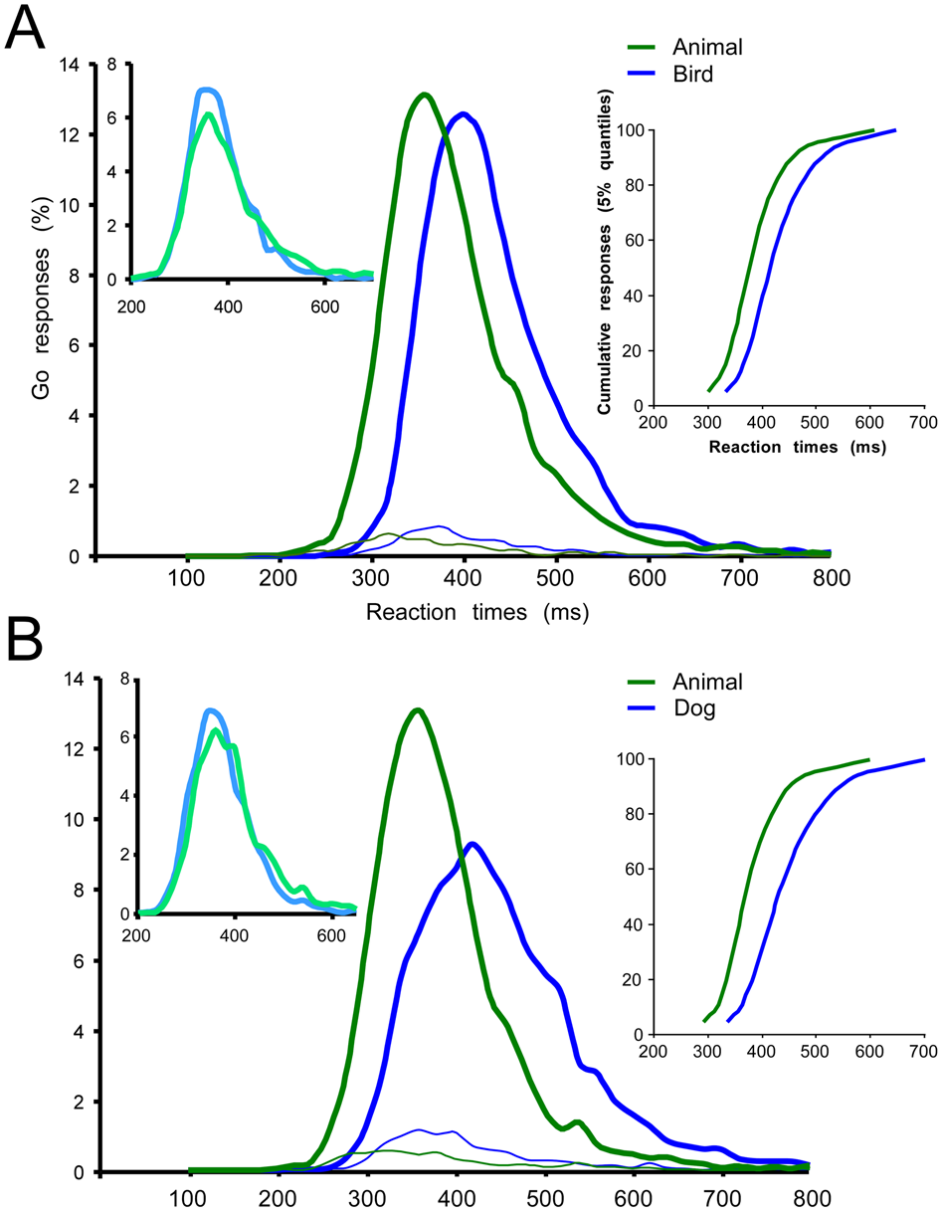
Reaction time distributions in the bird (A) and in the dog (B) experiments on correct (thick lines) and incorrect (thin lines) trials, calculated with 10 ms bin width. Reaction time distributions were computed separately in the superordinate level task (green curves) and the basic level task (blue curves). A and B: left insert correspond to RT distributions within the superordinate animal/non animal task for bird (dog) animal target (blue curve) and for other animal targets (green curves); right insert: vincentization of individual results (5% quantiles) in the superordinate (green curves) and the basic level (blue curves) categorization tasks.

Concerning the speed of responses, the animal control task was performed with comparable mean RTs in the Bird and the Dog experiments (394 ms and 386 ms respectively). In both control tasks, the pictures of birds and dogs were categorized faster than the pictures of other animals. Birds were categorized as animals with a mean RT of 385 ms (402 ms for all other animals; t-test, p<0.01) and dogs with a mean RT of 377 ms (394 ms for all other animals, t-test, p<0.01). These differences are accounted for by some very long latency responses recorded on some non-bird or non-dog animal target images that needed long processing times to be analyzed [21]. In contrast, the very first responses appeared at the same latencies (Fig 2). To evaluate the RTs of these earliest responses, we compute the minimal processing time (MinRT) that corresponds to the first time bin in the RT distribution from which correct responses significantly outnumber false alarms [22]. This MinRT reflects the shortest input-output processing time required by the system in a given task. In both control A/nA tasks the MinRT was the same for bird (or dog) photographs compare to all other types of animals (Bird experiment: 270 ms for birds and for other animals; Dog experiment: 260 ms for dogs and for other animals).

This control task shows that the very varied set of bird and dog images selected in these experiments were analyzed as any other animal image in the control A/nA task. If anything, they might be slightly easier to process as they were categorized on average 10 ms faster, with fewer long latency responses and even with a higher percentage of correct responses (+3%) in the case of bird photographs.

### Performance at the basic level (Bird/NonBird and Dog/nonDog)

#### Accuracy

**Figure 3:**
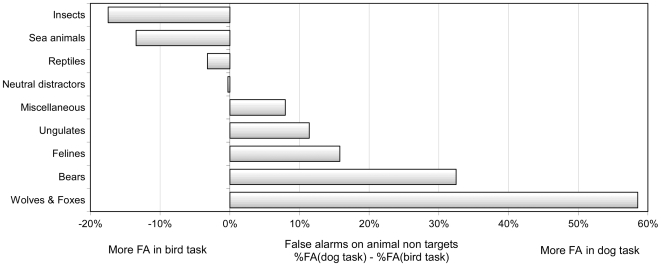
Comparison of false alarms elicited by different subgroups of animal non-targets depending on which animal was the “basic level” animal target. Rate of false alarms in the bird task were subtracted from rate of false alarms in the dog task. The results, expressed in absolute value, are reported on the left when more FA were performed in the bird task and on the right side when more FA were performed in the dog task. As an example, the value of 32.5% for bears on the right side is the result of subtracting 38.5% (false alarm rate for bears in the Dog task) and 6.0% (false alarm rate for bears in the Bird task).

When performing the categorization task at the basic level, subjects scored an average of 95.6% with birds and 92.6% with dogs. Considering only go responses towards targets, accuracy scores were slightly lower when subjects categorized birds as birds (basic level: 97.2%) than as animals (superordinate level: 98.5%; χ2, p<0.05; paired t-test, p = 0.001, t = 3.961). The same effect was observed with dogs that were categorized less accurately at the basic than at the superordinate level (94.7% vs. 96.8%; χ2, p<0.05; paired t-test, p = 0.014, t = 2.723). As in the superordinate A/nA tasks, subjects were better at responding on targets than at ignoring non-targets in both basic level tasks (97.2% vs. 94.0% in the Bird task, paired t-test, p = 0.005, t = 3.181, 94.7% vs. 90.5% in the Dog task; χ2, p<0.05, paired t-test, p = 0.028, t = 2.407). Half of the non-targets pictures were neutral (non-animal) images, but the other half were animals and it is important to specifically look at performance on these non-targets that belong to the same superordinate category. Indeed, the false alarms were mainly elicited by animal non-targets. In the Bird task, 90% of the false alarms were induced by pictures of non-bird animals, a proportion that reached 95% with non-dog animals in the Dog task. As for neutral non-animal photographs, they were ignored with a very high degree of accuracy (99% correct or over). Interestingly, depending on the basic level categorization task (bird or dog), some animal categories were more likely to induce false alarms. Figure 3 illustrates for different categories of non-target animals, the differential proportion of false alarms observed between the two basic level tasks. Insects and sea animals elicited numerous errors in the Bird task but were correctly ignored in the Dog task; the opposite was true for animals such as bears and felines.

#### Speed

Mean RTs in the two basic level categorization tasks were 434 ms in the Bird task and 452 ms in the Dog task. They were thus considerably longer than in the superordinate A/nA tasks; globally, subjects were 40 ms slower to categorize birds as birds and 65 ms slower to categorize dogs as dogs, even though they were processing the same sets of images at both basic and superordinate levels. As illustrated in figure 2, this effect was not limited to mean RTs and the whole RT distributions were shifted towards longer latencies in the basic level tasks. Early responses were thus also affected and MinRT increased by 50 and 40 ms respectively in the Bird (300 ms) and in the Dog (290 ms) tasks compare to the A/nA tasks (Fig 2).

But in such analysis of global RT distributions, the fastest subjects are always weighting more than slower ones for responses observed at short latencies. In order to better analyze the performance, we calculated the vincentized distribution of reaction times [23], [24]. The latencies of correct responses are ordered for each subject and processed by successive 5% quantiles. In the global performance of the group of subjects, each 5% quantile is defined as a weighted average of the corresponding quantile (mean RT of correct go-responses) of each subject of the group. By using this procedure, all subjects have the same influence along the axis of RT values. The vincentized global performance is illustrated in figure 2 and clearly shows the increase in reaction time needed to recognize a bird or a dog at the basic level of categorization. The curves computed for each task are parallel, showing that the additional processing time is stable for all responses regardless of their latencies even for difficult animal-target photographs; those that reliably induce long latencies responses in an animal categorization task [21].

### Implications

The two experiments in this first experimental series challenge the idea of a basic level advantage, at least in the case of animal categories. At the very least, the data are incompatible with a two-stage process in which access to superordinate animal representation follows basic level animal representations. In a go/no-go visual categorization task using complex natural scene photographs, subjects are much faster (40–65 ms in average) at categorizing animals than birds or dogs. Subjects appear to “spot” the animal before the bird (or dog). This performance speed is associated with a similar or even better (Dog experiment) accuracy at the superordinate level. This result is clearly at discrepancy with the large set of previous data demonstrating an advantage to access the basic level both in terms of speed and accuracy. The difference could come from the fact that we used “blocked trial” procedures in which subjects had to concentrate only on one category. However, such a block procedure would allow subjects to rely on an optimal strategy so that a given “favored” level of object representation should appear even more strongly in terms of performance. Some of the 40–65 ms speed advantage observed here in favor of the superordinate level could be due to the use of “natural” images. Virtually all studies reporting a speed advantage to access the basic level of categorization have used single isolated drawings/objects. Using drawings of full scenes, Murphy et al. [10] showed that the basic level advantage measured with objects seen in isolation is reduced when they are embedded in scenes. The use of natural scenes in our protocol might have reduced the basic level advantage, but natural scenes are the kind of stimuli our perceptual system has to deal with in daily life, so that they are more biologically pertinent to address the structural organization of perceptual categories.

When using objects in context, superordinate decision could be partly based on simple image statistics as shown for animals, people and vehicles [25]. However, in the present study, animal targets included a large number of stimuli for which simple image statistics would have predicted the presence of an animal only with low accuracy according to examples shown by Torralba and Oliva [25]. Moreover, non-target photographs included people in natural or man-made contexts that the subjects had to ignore. Object/context congruency has also been shown to interfere with animal superordinate fast categorization [26]. If object/context congruency also influences basic level categorization, this could explain the larger effects seen on accuracy and RT in the dog task as birds were mostly shown in natural contexts, whereas dogs could be equally presented in natural or man-made environments.

As mentioned in the introduction, another explanation that takes into account the task differences in terms of lexical requirements could explain the advantage shown here for the superordinate level. In their vast majority, previous studies reporting an advantage at the basic level used tasks that required a lexical access in addition to the visual processing of objects (category verification tasks, lexical priming, category naming, lexical input to switch target category at each trial, etc). In such tasks, response latencies are over 600 ms and more often seen around 800 ms or even 1000 ms. At these long latencies, the present study shows that perceptual representations are refined enough to allow basic level categorization. Thus, because the basic level words “dog” and “bird” are most commonly used to label such an animal, they could well be accessed faster and the speed advantage described in our study would be wiped off. The revealed architecture of category hierarchy might then be derived from lexical constraints whereas, in our study, it would reflect more specifically the functional architecture of the visual system in the early progressive shaping of perceptual representations as proposed in the Parallel Distributed Processing (PDP) theory [19], [20]. Object representations would emerge from perceptual, motor and linguistic representations. Broad categories would be activated before tuning to more specific representation, but although the word “bird” would be activated after the word “animal”, it would become fully activated much faster. A clear prediction from the PDP theory is that there should be an advantage for superordinate categories when subjects are encouraged to make fast decisions, and indeed Rogers and Patterson found better accuracy at the general level for fast decisions in a verification task [20]. Our results based on fast visual categorization clearly support the PDP theory and point towards an early temporal window during which categorization is possible at the superordinate level but not at the basic level. In fast visual categorization tasks, object superordinate representation might even not be conscious. Subjects can categorize stimuli appearing in very far periphery [27]; they can process two and up to four simultaneously presented stimuli [22], [28] and they can perform the task at no cost when their attention is focalized elsewhere in a dual-task paradigm [29].

In the visual domain, the superordinate level may not constitute an abstraction from basic levels as previously proposed [4], [7], but rather the rudimentary level at which some coarse object representations can be accessed with early crude processing of visual information. This idea is very close to the coarse to fine functional architecture of the visual system proposed by Schyns et al. [30], [31], [32], [33]. Previous works including neuronal responses in monkeys [34], MEG in humans [35] or psychophysical data [36], [37] support this model in the domain of face processing with an early stage for face categorization and a later stage for identifying individuals or expressions. During scene reconstruction along the visual pathway, the early processing of the highest saliency locations (strongest contrasts) may be sufficient to infer the coarse structure of salient objects and perform a task at the superordinate level. In contrast, the system would need additional processing of incoming information to reach a more detailed object representation and access basic level categories. The early activation of object representations that belongs to a given superordinate class will also have the advantage of narrowing the search domain for further categorization or identification. With such an interpretation, we are again in line with the PDP theory that proposes a progressive tuning to reach more and more specific object representations. This progressive tuning is supported in our data by the fact that the very same bird and dog photographs were classified as animal with a higher accuracy than at the basic level, and that false alarms in the basic task were triggered mainly by animal pictures. The 40–65 ms additional processing time when subjects are looking for birds or dogs prove sufficient to avoid making false alarms on non-targets that do not contain animals. The vast majority (90–95%) of false alarms were induced by the 50% non-targets that contained an animal. It is worth noting that all animals can induce false alarms even though the specified target (bird or dog) induced a clear bias towards some specific animal species. This strongly suggests an early widespread activation of all kinds of animal representations. Extracting selectively those representations that are more specific to a precise target category would take additional processing. In other words, perceptual coarse representations of the superordinate animal category might be available earlier than the finer representation necessary to take a basic level dog or bird decision. Our data do not provide information about the nature of “animal representations”; they might just be based upon a set of animal features. Indeed the absence or presence of some typical animal features (eyes, mouth legs) can modulate response latency in our fast visual categorization task [38]. The role played by animal features might be crucial at the basic level. When looking for a bird or a dog, top down influences could modify the visual system expectations by presetting the type of pertinent animal features that subjects should look for. As an illustration, errors in the Bird/non Bird task were typically made on insects and fish that share features such as lateralized eyes, wing-like structures and the absence of ground support (in the air or underwater). In contrast, errors in the Dog task were observed mainly on canines (foxes and wolves), bears or felines (Fig 3). Birds and dogs are not at the same level in the animal taxonomy. Whereas birds constitute a class on their own (Avian), dogs are only a species within the mammalian class. Thus, birds have some very specific visual features (feathers, wings, beaks, side eye location, aerodynamic shapes) and functional characteristics (they usually fly). On the other hand, dogs are prototypical mammals and share numerous characteristics with a large number of them such as having 4 legs, 2 ears, frontal eye location, a body covered with fur… The expected consequence is that more visual features are diagnostic for birds than for dogs, so that they should be more easily distinguished from other animals than dogs. Conversely the accuracy on the Dog task was lower than in the Bird task (92.6% vs. 95.6%, χ2, p<0.05; paired t-test, p = 0.028, t = 2.409) and the required additional processing time was longer (65 ms compared to 40 ms).

The physical distance between targets and non-targets is of major importance. Bowers and Jones [3] compared basic level categorization between dissimilar basic categories (dogs vs. buses) or very similar categories (dogs vs. cats). They found that their first task was easier to perform (accuracy and speed), although it has to be noted this task could be done on the basis of coarse representation of superordinate categories (animal vs. vehicles). Although less stressed by Grill-Spector and Kanwisher in their 2005 paper, when looking at speed of processing for basic categories, the non-target exemplars belonging to the same superordinate category were chosen by the authors to be very different: when dogs were targets, only birds and fish were used as non-targets and when guitars were targets no other string instruments were used as non-targets. Considering the errors reported above, the absence of exemplars sharing visual properties with the targets must have had a considerable impact on the speed of processing.

### Experimental series 2. Basic categorization: influence of non-target set

The second experimental series was designed to analyze in detail the effect of using different ratios of non-target images belonging to the target superordinate category. In the first experimental series, half of the non-targets were neutral and the other half contained non-target animals. In this second experimental series, we compared this situation with two other conditions: (1) a more difficult situation in which the proportion of non-target animal was increased to 100% and (2) an easier situation in which all non-target images were neutral. Expected results were a drop of performance in condition (1) and an improved performance in condition (2). In fact, in condition (2) the task can be solved at a superordinate level because all non-targets were non-animal images, thus performance should be similar to the animal/non-animal task. However, since the target domain is much more restricted as similarity is greater within the basic domain than within the superordinate domain, an improved performance could also be expected.

### Performance in the superordinate control task (A/nA)

In the superordinate categorization task, the scores (global accuracy: 94.8% and mean RT: 395 ms) reached by the group of 14 new subjects were similar to those reached by the group of 16 subjects tested in the preceding experiment (95.5% and 386 ms). Here again, subjects had a tendency to be better at responding on animal targets (go responses) than at ignoring non-targets (no-go responses) 96.4 vs. 93.1% (χ2, p<0.05, paired t-test, p = 0.054, t = 2.121). Dog photographs were categorized as “animal” with a slightly better accuracy (97.3%) than non-dog animal pictures (95.5%) (paired t-test, p = 0.014, t = 2.828) but with a mean and median RT that were about 15 ms longer (mean RT 403 ms vs. 388 ms, paired t-test, p = 0.003, t = 3.664; median RT 384 ms vs. 369 ms). In this experiment, the group of subjects showed a speed accuracy trade-off categorizing with a slightly higher accuracy but slower speed the set of dog pictures.

### Effect of non-target set composition on performance in the basic level tasks

**Figure 4:**
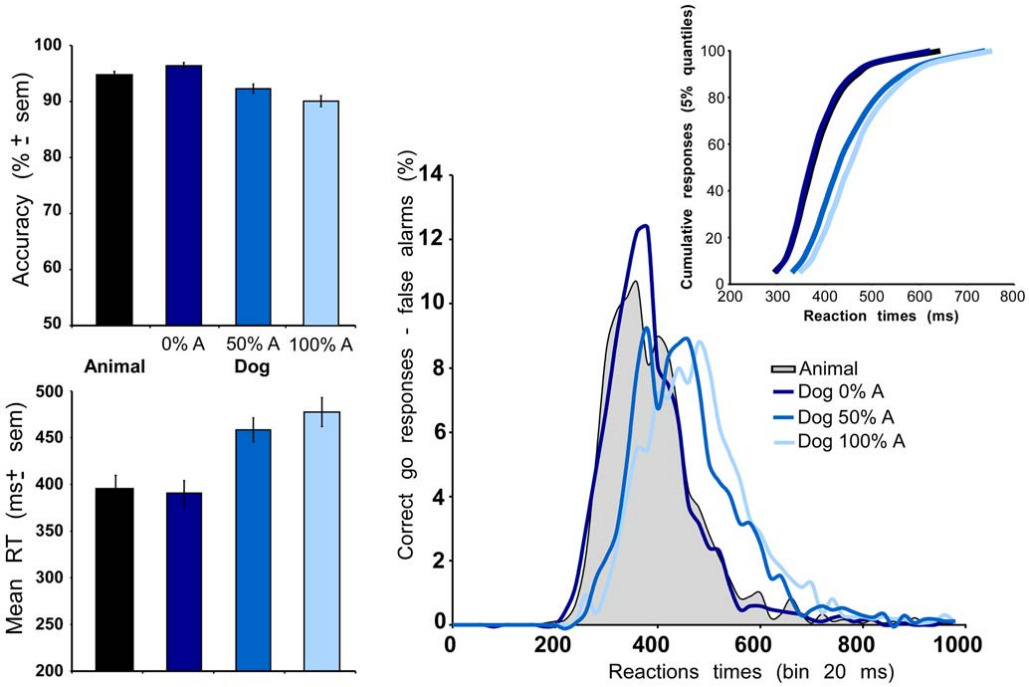
Performance in the superordinate categorization task (black curves and black or gray histograms) and in the categorization task at the basic level (dogs) with non-targets including 0%, 50% or 100% other animals (dark, middle and light blue curves and histograms). Left: global mean accuracy and standard deviation on the mean expressed in % correct, Global mean reaction times and standard deviation on the mean expressed in ms. Right bottom: histogram of reaction times computed for each experimental condition by subtracting false alarms from correct go-responses in each 10 ms time bin. Right left: vincentization of individual results separately in the superordinate level task (black curves) and the basic level task (blue curves). Right insert: vincentization of individual results (cumulative responses, 5% quantiles) in each of the experimental conditions. Note that for the two conditions: superordinate animal categorization task and Dog basic level task with no other animal non-target, curves are totally superimposed.

The aim here was to compare human ability to categorize dogs as dogs when the non-target set included an increasing proportion of non-dog animals (Fig 4). Global accuracy was indeed dependent on the non-target set. With an increasing ratio of non-dog animal to ignore (0%, 50%, 100%), accuracy decreased from 96.4% correct to 92.3% and 90.1% (paired t-test, 0%–50%: p = 0<0.001, t = 6.435, 0%–100%: p = 0<0.001, t = 10.632, 50%–100%: p = 0.006, t = 3.306). This accuracy decrease was not obvious on correct go-responses towards dog-targets (respectively 95.9%, 95.5% and 94%, although a paired t-test indicated a significant difference when comparing 0%A and 100%A condition: p = 0.026, t = 2.509). On the other hand it affected significantly the rate of false alarms triggered by non-targets. The proportion of correct no-go responses was very high in condition Dog0%A (96.9%) and decreased as soon as non-dog animal were introduced in the non-target set (89.1% and 86.2% respectively in condition Dog50%A and Dog100%A, paired t-test, 0%A–50%A: p<0.001, t = 6.162, 0%A–100%A: p<0.001, t = 6.790, 50%A–100%A: p<0.05, t = 2.600).

Whereas accuracy on dog targets was very similar in the 3 conditions, the speed of response was strongly affected. In the Dog0%A, mean RT on correct go-responses towards dog-targets was 391 ms, it went up to 459 and 478 ms in conditions Dog50%A and Dog100%A (paired t-test, 0%A–50%A: p<0.001, t = 7.367, 0%A–100%A: p<0.001, t = 9.430, 50%A–100%A: p<0.005, t = 3.720). The same effect was observed when considering median RT (respectively 375 ms, 439 ms and 464 ms). Closer analysis of RT distributions clearly shows a shift towards longer latencies when the non-target set includes non-dog animals (Fig 4). In fact, when the non-target set does not include any animal (superordinate task or Dog0%A condition) the two distributions are remarkably similar. On the other hand, processing a dog as a dog when non-targets include other non-dog animals requires more processing time. The results are not due to the fastest subjects as the vincentization of the RT by 5% quantiles of individual RTs shows the same effect (Fig 4).

Thus a large effect was seen with the introduction of 50% non-targets belonging to the same superordinate category with a false alarm rate increase of 7.8% and a median RT increase of 64 ms. Further increase (from 50 to 100%) just strengthened this effect with an additional increase in false alarms and median RT (2.9%, 25 ms).

**Figure 5:**
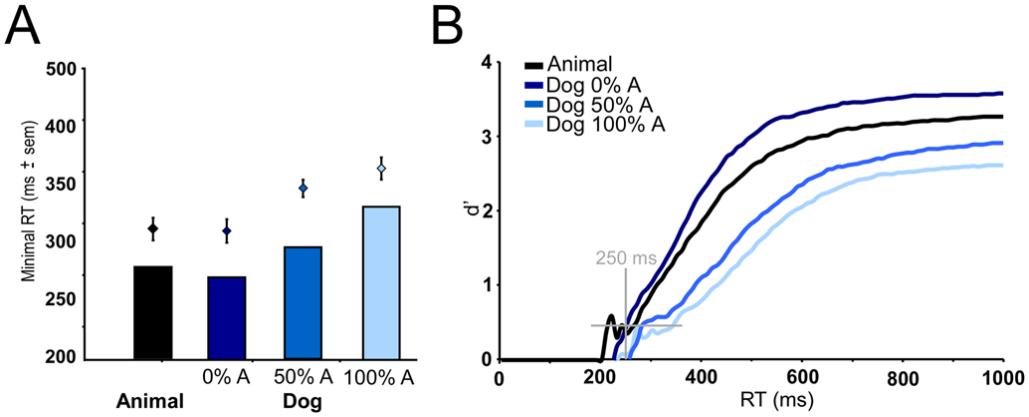
Performance in the superordinate categorization task (black curves and histograms) and in the categorization task at the basic level (dogs) with non-targets including 0%, 50% or 100% other animals (dark, middle and light blue curves and histograms). A. Minimal reaction time determined as the first 10 ms time bin for which correct responses significantly exceed errors (targets and non-targets were equally likely) and processed on cumulated data (histograms) or as the mean of individual data (diamonds with standard deviation on the mean). B. Cumulative d' curves using signal detection theory sensitivity measures were plotted as a function of time with 10 ms time bins. Cumulative number of hits and false alarm responses were used to calculate dV =  zhits - zFA at each time point where z is the inverse of the normal distribution function (Macmillan & Creelman, 2005). d' curves corresponding to the time course of performance give an estimation of the processing dynamics for the entire subject population. The shortest minimal reaction time shown on the left is indicated on the d' curves to draw attention on the shift of performance for the shortest “meaningful” behavioral responses.

With the increase in false alarms, computing MinRT and d' curves is of particular importance. The MinRT value increased with the ratio of animal non-targets (0%, 50%, 100%) regardless of the analysis performed to compute the MinRT (mean of individual MinRTs: 306, 348, and 367 ms; MinRT processed on group overall performance: 250, 280 and 320 ms). In all cases, MinRT computed for the superordinate task appears very close to MinRT at the basic level of categorization in the Dog0%A condition (309 ms or 260 ms compared to 306 ms or 250 ms). MinRT corresponds to the first latency at which correct responses significantly outnumber false alarms, but it is also interesting to follow performance as a function of response latency. For each task condition, we processed the corresponding dynamic d' curves (Fig 5). A slight advantage is seen for the Dog0%A condition over the superordinate task, but there again the results show that to reach the same d' values when 50% of the stimuli to ignore are non-dog animals, an additional processing time is clearly needed and even increased when this ratio reaches 100%.

### Categorization of dogs at superordinate and basic levels with neutral non-targets

Categorization performance at the superordinate and basic levels are compared with identical sets of target images, and sets of non-targets as varied in both conditions, but the search space for possible targets is reduced strictly to dogs at the basic level whereas it includes all other animals in the superordinate task. In such condition, one might have expected that a speed advantage would clearly appear for categorization at the basic level. But this was not the case as RT distributions with or without vincentization for all subjects performing either at the superordinate or at the easiest basic level condition, always resulted in early responses produced at the same latencies and with the same accuracy in both conditions. Plotting the dynamic cumulative d' curve (Fig 5) is the only analysis that revealed a slight advantage for performance at the basic level when the non-target set did not contain exemplars of the same superordinate category: an advantage that might result from the lower proportion of false alarms (A/nA: 6.9%, dog0%A: 3.1%).

This second experimental series shows that we can effectively “spot” the animal before the dog and that more processing time is needed to access basic level categories. It is only when the task was rendered easier by removing all non-target images belonging to the same animal superordinate category that a very little advantage for basic level was seen in terms of false alarms reduction. Note that in the absence of non-targets from the same superordinate category, the task could be performed indistinctively at the basic or at the superordinate level.

## Discussion

The more abstract (superordinate) visual representation appears to be available earlier with subsequent refinements needed to allow categorization at the basic levels. This might reconcile the different claims made recently. Object categorization at the superordinate level might indeed be as fast as object detection, and this would explain why Grill-Spector and Kanwisher [1] found no processing speed difference. Their sets of stimuli were close to the dog0% condition because they had chosen to include only fish and birds as non-target animals: animals that do not have much similarity with mammals in general and did not induce many false alarms in the dog basic level categorization task. This was also why Bowers and Jones [3] found that categorization was as fast as detection when using what they call “easy” basic categories (dogs vs. buses) but needed more processing time for a more difficult categorization (dogs vs. cats). Their easy categorization is actually a superordinate categorization task with a search space restricted to one basic level category. In this task, the distance in terms of visual similarities is both quite short between targets, as they belong to the same basic category and quite large between targets and non-targets as they belong to different superordinate categories. Thus performance for such tasks could just rely on a crude representation of the superordinate categories which can explain why categorization is still possible well over chance at very low contrasts [33] or at very large eccentricities [27]. They may also rely on some diagnostic intermediate key features [38], [39]. Indeed, the same advantage for superordinate categories has been found for categorizations of scene contexts -manmade vs. natural- over categorization at the basic level: sea, mountains, indoor, outdoor [40], [41] and contextual categorization might rely on image simple statistics or relatively low resolution sketch [25], [42]. This interpretation would be in keeping with the “coarse to fine” hypothesis [30], [41], [43], [44].

It might be that, because they are so biologically relevant, animals constitute a very special category. Indeed, in the infero-temporal lobe of monkeys passively looking at natural photographs, response patterns of neuronal populations can reflect object category structure. Animate and inanimate objects have been shown to create distinguishable clusters in the population code [45] so that the activation of one or the other cluster could provide a basis for a response at the superordinate level. Moreover, using a phenomenon called “change blindness”, New et al. [46] showed that human subjects were both faster and more accurate at detecting changes concerning animals than vehicles, buildings, plants or tools. They concluded that the monitoring advantage for animals could reflect ancestral priorities.

Although animals might constitute a very special superordinate category, there are some reasons to believe that the results found in the present study might generalize to other categories. First we already observed an advantage for global domain categories using scene categories: there was a speed advantage for distinguishing between natural and man-made environments whereas reaching a decision at more detailed levels (sea, mountain, indoor or outdoor environments) required a longer processing time [41]. As far as object categories are concerned, our results will have to be replicated with an artifactual category. However, it has already been shown that fast visual categorization was as fast and as accurate for “vehicles” as for “animals” [13]; here again it would be very surprising if human performance could be faster for a basic category of vehicles. Moreover in their 2007 study, Rogers and Patterson were testing both animals and vehicles when they showed an accuracy advantage for superordinate over basic levels when subjects had to perform fast responses.

Our results do not support the hierarchical model in which visual stimuli are first classified at the basic level and need additional late information to reach super- or sub-ordinate levels as suggested by Jolicoeur and coll. [7]. Instead they show an early temporal window during which the accuracy of subjects increases very fast for response at superordinate level whereas responses at the basic level have not been initiated yet. The ability to extract early coarse information about categories is documented by a recent study [47] showing that early synchronizations in brain activity (around 100 ms) might be sufficient for crude (superordinate) representation of objects in scenes, whereas entry level categorization would depend upon later brain activity (200–400 ms). The extraction of early coarse information is also supported by other studies. When subjects are presented with grayscale photographs masked after a variable (27–500 ms) duration and asked to report with accuracy what they had seen, the reporting of sensory- or feature-level information of a scene consistently preceded the reporting of the semantic-level information [48]. Moreover “animals” were reliably reported with shorter image presentations than “birds”, “dogs” or “cats”. Dell'Acqua & Grainger [49] also concluded that only gross semantic information related to superordinate categories could be extracted using very briefly presented, pattern-masked picture as primes. Such primes were efficient for word or picture categorization but not for word reading. Finally global domain categories might develop before true basic level ones in children [11].

There are two main explanations for why the superordinate level appears to be accessed before basic or subordinate level. The first one would include a two-stage process, in which superordinate representations need to be accessed before basic levels. But if there is no need for that first step to be completed, a second alternative would postulate parallel access to all representations with a faster access to the superordinate level as suggested in the PDP theory. The perceptual representation of the broad “animal category” would be accessed early before tuning to more specific representations such as bird and then canary.

When task performance also requires a lexical/semantic access, the necessary integration of multimodal information might need more time to develop. A recent MEG study [50] suggests that perceptual categorization precedes semantic-conceptual categorization. In the human medial temporal lobe, neurons responding to a particular class of visual objects (such as animals) can start firing as early as 220 ms [51]. More sophisticated neuron assemblies with multimodal object representations have also been reported [14]. They respond to varied photographs of a specific visual object, its caricature and even the letter string of its name, but they have much longer latencies, typically in the 300–600 ms range.

Although we cannot infer the nature of the early “animal” representations from our results, it might depend upon animal diagnostic features that characterize the targets relatively to the non-targets as argued by others [52]. Indeed in the second experimental series, the basic level is accessed as fast as the superordinate level when the non-target images do not include animals. In such case any animal feature would belong to a dog target! When non-target images include non-dog animal, the top down presetting of the visual system must be specifically tuned to dog features while ignoring non-dog animal features… Such top down modulation of pertinent features can also be modulated by expertise that would be able to play a critical role.

Indeed, another important factor that should be considered in categorization experiments is the personal knowledge of the subjects and their expertise with the object categories used as targets. Whereas subjects needed more time to categorize dogs and birds at the basic level, it is worth noticing that they can categorize human beings as fast as animals [53], thus no additional processing time is needed for human-targets with an accuracy that is even better! It might be that, as often claimed, humans are a entry level category on their own. Alternatively, it could also be the result of our extreme expertise with human beings. Manipulating category structures and boundaries is not a new idea and has previously been performed to explain typicality and expertise effects [5], [8], [54]. The underlying hypothesis is that training on a particular set of stimuli can possibly modify the representations in the inner visual processing and facilitate recognition by increasing encoding speed. Together with specific lexical labels, experts in birds and dogs might build visual representations of birds and dogs based on early available visual information. This needs to be investigated further although expertise might have more effect on the distance between basic and subordinate representation than the distance between basic and superordinate representations.

To sum up, the present data show that visual representation of superordinate category might be accessed first and that more detailed representations would require more processing time. As soon as one sees an object, one might know which superordinate category it belongs to. The time required to access visually perceived basic level categories is a more complicated story and probably depends both on expertise and on similarity between target and non-target exemplars.
